# Reduction of circulating innate lymphoid cell progenitors results in impaired cytokine production by innate lymphoid cells in patients with lupus nephritis

**DOI:** 10.1186/s13075-020-2114-5

**Published:** 2020-03-29

**Authors:** Seungwon Ryu, Eun Young Lee, Dong Ki Kim, Yon Su Kim, Doo Hyun Chung, Ji Hyung Kim, Hajeong Lee, Hye Young Kim

**Affiliations:** 1 Laboratory of Mucosal Immunology, Department of Biomedical Sciences, Seoul National University College of Medicine, Seoul, 03080 South Korea; 2 Division of Rheumatology, Department of Internal Medicine, Seoul National University Hospital, Seoul, South Korea; 3 Division of Nephrology, Department of Internal Medicine, Seoul National University Hospital, Seoul, South Korea; 4 Kidney Research Institute, Seoul National University College of Medicine, Seoul, 03080 South Korea; 5 Department of Pathology, Seoul National University College of Medicine, Seoul, South Korea; 6 Laboratory of Immune Regulation, Department of Biomedical Sciences, Seoul National University College of Medicine, Seoul, South Korea; 7 College of Life Sciences and Biotechnology, Korea University, Seoul, South Korea; 8 Institute of Allergy and Clinical Immunology, Seoul National University Medical Research Center, Seoul, South Korea

**Keywords:** Lupus nephritis, Autoimmune kidney disease, Innate lymphoid cells (ILCs), Cytokine

## Abstract

**Background:**

Innate lymphoid cells (ILCs) play an essential role in maintaining homeostasis; however, they can also cause chronic inflammation and autoimmune disease. This study aimed to identify the role of ILCs in the pathogenesis of lupus nephritis (LN).

**Methods:**

The percentage of ILCs within the peripheral blood mononuclear cell (PBMC) population and urine of patients with LN (*n* = 16), healthy controls (HC; *n* = 8), and disease controls (ANCA-associated vasculitis (AAV; *n* = 6), IgA nephropathy (IgAN; *n* = 9), and other glomerular diseases (*n* = 5)) was determined by flow cytometry analysis. In addition, ILCs were sorted and cultured with plasma from LN patients or HC to elucidate whether the reduced population of CD117^+^ ILCs observed in LN was due to changes in the ILC progenitor population.

**Results:**

The percentage of total ILCs and CD117^+^ ILCs in LN was significantly lower than that in HC. The percentage of cytokine-secreting ILCs was also lower in LN; however, when the disease stabilized, cytokine production was restored to levels similar to those in HC. The increase in the number of exhausted ILCs (cells unable to secrete cytokines) correlated positively with disease activity. When CD117^+^ ILCs were cultured with LN plasma, the number of CD117^+^ ILCs fell, but that of other ILC subsets increased.

**Conclusions:**

The percentage of CD117^+^ ILCs and the capacity of ILCs to secrete cytokines fell as LN severity increased, suggesting that an inflammatory environment of LN induces persistent differentiation and exhaustion of ILCs.

## Introduction

Lupus nephritis (LN) is a renal inflammation caused by systemic lupus erythematosus (SLE). SLE is characterized by manifestations of various organs and the presence of autoantibodies such as anti-double-stranded DNA (anti-dsDNA) and anti-Smith antibodies [1]. The organ most commonly affected is the kidney. Moreover, invasion of the kidney is a major prognostic factor for long-term morbidity and mortality for patients with SLE [1]. Genetic and environmental factors contribute to the pathogenesis of LN; however, the exact mechanism is unclear. Genome-wide association studies of patients or mouse models of lupus nephritis suggest that protection from autoantigens (e.g., nucleic acids or histone proteins) induced immune reactions is compromised at various checkpoints [2, 3]. Renal inflammation, mainly glomerulonephritis, persists with autoantibodies that cross-react with endogenous glomerular antigens. In addition, deposition of immune complexes within glomeruli recruits immune cells via complement activation, thereby aggravating the inflammation [4]. Current treatments for severe LN are based on nonspecific immunosuppression by high-dose glucocorticoids along with cytotoxic agents such as cyclophosphamide or mycophenolate mofetil, all of which have dose-limiting side effects [5]. Thus, the development of novel therapeutic targets based on the specific pathogenesis of the disease is needed [6, 7].

Innate lymphoid cells (ILCs) are a newly discovered type of immune cell that is characterized by antigen-independent activation. Although they do not react in an antigen-specific manner, ILCs resemble T cells in that they share key transcription factors and produce cytokines. Group 1 ILCs (ILC1s) including NK cells express T-bet and produce IFN-γ and TNF-α. Group 2 ILCs (ILC2s) express GATA3 and produce mainly type 2 cytokines such as IL-5 and IL-13. Group 3 ILCs (ILC3s) express RORC and produce IL-17 and/or IL-22 [8]. Conventionally, human and mouse ILCs are classified according to the expression of surface markers; Lin^−^ (Lineage (Lin) markers; a collection of surface markers of immune cells, not those of ILCs) CD127^+^ lymphocytes. Among ILC subsets, Lin^−^ CD127^+^ CD117^+^ lymphocytes are considered as ILC3s; however, the current thinking is that circulating CD117^+^ ILCs are mainly progenitor cells rather than ILC3s [9, 10].

Although ILCs play roles in several autoimmune diseases, including rheumatoid arthritis, juvenile inflammatory arthritis, spondyloarthritis, and systemic sclerosis [11–14], their role in autoimmune kidney diseases is unclear. Recently, ILC2s were identified as a major ILC population in the healthy kidneys of mice [15]. These cells are located in both glomerular and tubulointerstitial areas following the vasculature of a mouse kidney [15, 16]. Work in murine models of renal disease suggests that ILC2s protect the kidney from toxic [15] or ischemic kidney injury [17, 18] by producing type 2 cytokines and amphiregulin. In addition, a murine model of LN revealed a reduced percentage of ILC2s in the injured kidney, but in vivo expansion of ILC2s (mediated by IL-33) ameliorated kidney inflammation; this suggests a protective role of ILCs [19]. However, human data supporting these results are scarce. Here, we examined the role of ILCs in LN and other autoimmune kidney diseases by analyzing ILC populations isolated from both peripheral blood and urine samples from LN patients, disease controls, and healthy controls (HC).

## Methods

### Subjects

We enrolled active LN patients and active disease controls when they received diagnostic kidney biopsy in the Seoul National University Hospital, Korea. Stable LN patients were enrolled during their outpatient department visits. Live kidney donors were included as healthy controls (HC) when they donated their kidneys altruistically. LN patients met the criteria of either the 1997 update of the 1982 American College of Rheumatology Revised Criteria for Classification of Systemic Lupus Erythematosus [20] or the 2012 Systemic Lupus International Collaborating Clinics Classification Criteria for Systemic Lupus Erythematosus [21]. The stable LN was defined as patients without any systematic manifestations of SLE and normal renal function. Disease controls included autoimmune kidney disease controls (anti-neutrophil cytoplasmic antibody (ANCA)-associated vasculitis (AAV) and IgA nephropathy (IgAN)) and other glomerular renal disease controls (minimal change disease (MCD) and focal segmental glomerulosclerosis (FSGS)). We obtained blood, urine, or renal tissues from patients with active LN, disease control, and HCs, whereas only blood samples from those with stable LN. Information on demographic factors, clinical manifestations, and laboratory tests was reviewed at the time of sample acquisition (Table 1).

**Table 1:** Characteristics of the study group

	Active LN (*n* = 16)	Stable LN (*n* = 10)	AAV (*n* = 6)	IgAN (*n* = 9)	MCD/FSGS (MCD, *n* = 4; FSGS, *n* = 1)	HC (*n* = 8)
Age (years)	31 (23–45)	53 (31.75–61.25)	52 (45.5–59.5)	45 (35.5–57)	53 (40.5–62)	49.5 (40.5–55.5)
Female, *n* (%)	15 (93.75)	10 (100)	4 (66.67)	8 (88.89)	3 (60)	6 (75)
WBC count, × 10^3^/mL	4.43 (3.32–6.54)	4.30 (3.67–4.89)	9.98 (6.89–15.37)	8.1 (7.36–8.35)	8.02 (6.28–10.01)	5.44 (4.56–7.77)
CRP, mg/L	0.15 (0.07–1.15)	0.05 (0.03–0.16)	2.21 (0.38–10.35)	0.03 (0.01–0.15)	0.04 (0.02–0.05)	0.02 (0.01–0.05)
Serum BUN, mg/dL	14.5 (12–18.75)	15.5 (12.25–22.25)	31 (21.75–58.75)	17 (14.5–27)	14 (11.5–23.5)	15 (11–16)
UPCR, mg/mg	2.93 (0.90–5.87)	0.21 (0.09–0.36)	2.16 (1.233–2.97)	1.6 (0.90–4.62)	9.55 (3.99–16.88)	0.05 (0.04–0.11)
Serum anti-dsDNA, IU/ml	512 (267.9–849.8)	6.7 (2–155.9)	N/A	N/A	N/A	N/A
C3, mg/dL	44.5 (39.5–59.75)	69.5 (47.75–84.75)	106.5 (93.75–112.3)	99 (85–124)	N/A	N/A
C4, mg/dL	8 (5.5–16)	10.5 (4.5–21)	25 (12.5–30.25)	28 (20.5–32.5)	N/A	N/A

The data are presented as the median and interquartile range (IQR) or as number (percentage)

*Abbreviations*: *LN* lupus nephritis, *AAV* ANCA-associated vasculitis, *IgAN* IgA nephropathy, *MCD* minimal change disease, *FSGS* focal segmental glomerulosclerosis, *HC* healthy control, *WBC* white blood cell, *CRP* C-reactive protein, *BUN* blood urea nitrogen, *UPCR* urine protein to creatinine ratio, *dsDNA* double-stranded DNA, *C3* complement component 3, *C4* complement component 4

### Cell isolation from peripheral blood, urine, and kidney tissue

Peripheral blood mononuclear cells (PBMCs) were isolated in a manner of density-gradient separation principles using Ficoll-Paque™ PLUS (GE Healthcare, Uppsala, Sweden) as previously described [22]. Urine cells were washed and resuspended in 40% Percoll™ (GE Healthcare, Uppsala, Sweden), which was layered onto 80% Percoll solution. Leukocytes from urine samples were obtained from the interface between two Percoll layers after centrifugation at 1800 rpm, 4°C for 25 min. A normal portion of nephrectomized kidney from renal cell carcinoma, used for comparing the characteristics of ILCs from kidney and urine, was mechanically and enzymatically dissociated for single-cell preparation as described previously [17].

### Flow cytometry and sorting of ILCs

Single cells from either blood, urine, or renal tissue were blocked with human BD Fc Block™ (BD Biosciences, NJ, USA). Cells were stained with fluorescent monoclonal antibodies for 30 min at 4 °C. For intracellular staining, cells were fixed and permeabilized with BD fixation/permeabilization solution kit (BD Biosciences, NJ, USA) for 20 min at 4 °C. Cells investigated for cytokine production were stimulated with PMA (100 ng/ml; Sigma-Aldrich, MO, USA)/ionomycin (1 μg/ml; Sigma-Aldrich, MO, USA) and incubated with GolgiStop™ (0.7 μL/ml; BD Biosciences, NJ, USA) for 4 h at 37 °C. Cytokines were stained with fluorescent monoclonal antibodies overnight at 4 °C. Antibodies used for flow cytometric analysis or cell sorting were as follows: lineage markers (anti-human CD3 (clone: UCHT1), anti-human CD19 (HIB19), anti-human FcεRIα (AER-37), anti-human CD49b (P1E6-C5), anti-human CD11b (ICRF44), anti-human CD11c (3.9), and anti-human CD14 (HCD14)), anti-human CRTH2 (BM16), anti-human CD117 (104D2), anti-human NKp44 (P44-8), anti-human PD-1 (EH12.2H7), anti-human IL-13 (JES10-5A2), anti-human IL-17A (BL168), anti-Annexin V and Streptavidin from Biolegend (CA, USA), and anti-human IFN-γ (B27) from BD Biosciences (NJ, USA). Flow cytometric data were collected by LSRFortessa X-20 (BD Biosciences, NJ, USA) and analyzed by FlowJo v10 (BD Biosciences, NJ, USA). Fluorescence-labeled ILCs were sorted with Aria III (BD Biosciences, NJ, USA).

### Immunofluorescence of kidney tissue sections

Kidney tissues were obtained from percutaneous needle biopsy in cold PBS. Tissues were mounted on Surgipath FSC22 Frozen Section Compound (Leica, IL, USA) and stored at − 80 °C until the section of the tissues. As the first step for staining, tissues were fixed with cold acetone. After blocking, primary and secondary antibodies were applied to tissue section and incubated for an hour at room temperature, respectively. Coverslip was mounted on the tissue slides after application of ProLong™ Diamond Antifade Mountant with DAPI (Life Technologies, OR, USA) applied to the tissue slides. The antibodies used for the staining were as follows: anti-c-Kit antibody (host: rabbit, polyclonal) from Biorbyt (Cambridge, UK), anti-CD3 antibody (rat, polyclonal) from Abcam (Cambridge, UK), and anti-rabbit AF594 (donkey, polyclonal) and anti-rat AF488 (donkey, polyclonal) from ThermoFisher Scientific (IL, USA). Slides were imaged by confocal microscopy, FV3000 (Olympus, Tokyo, Japan) and analyzed with FV10-ASW 4.0 Viewer (Olympus, Tokyo, Japan).

### Culture of circulating CD117^+^ ILCs

For whole blood immune cell cultures, 2 × 10^6^ PBMCs were cultured in plasma from healthy controls or LN with recombinant human IL-2 and IL-7 (40 ng/ml each; R&D Systems, MN, USA) for 24 h. To test the role of CD117^+^ ILCs as ILC progenitors in LN condition, CD117^+^ ILCs were sorted, and 200–300 cells were cultured with plasma from healthy controls or LN for 8 days. 2.5 × 10^3^ OP9 cells (ATCC, VA, USA) were seeded on a 96-well round-bottom plate a day before CD117^+^ ILC culture, as previously described [9]. Recombinant human IL-2 and IL-7 were added on each well every 3 days. To verify the contribution of IL-1 receptor signaling in the differentiation of blood ILC progenitors, human IL-1R1 blocking antibody (2 μg/ml; R&D Systems, MN, USA) was added to the whole blood immune cell cultures with HC or LN plasma as the whole blood immune cell culture assay above. Serum level of human IL-1β was quantified by ELISA (R&D Systems, MN, USA).

### Statistical analysis

The percentages of ILCs were compared between various renal disease groups or different disease activity. Two unpaired-groups were compared using Student’s *t* test for the parametric measure or Mann-Whitney test for nonparametric measures. Paired Student’s *t* tests were used to calculate the differential effect on blocking IL-1 receptors on blood ILCs. For correlation analysis, the Pearson correlation for a parametric measure or Spearman correlation for a nonparametric measure was used. The determination of the normality of data was based on the Shapiro-Wilk test. Graphs were displayed with median, and the first and third quartiles (Q1–Q3). The significance level was 0.05 for two-tailed tests. Statistical analysis was performed with GraphPad Prism 7 (GraphPad, CA, USA).

## Results

### The percentage of CD117^+^ ILCs in blood and urine from patients with LN is lower than that in controls

**Fig. 1:**
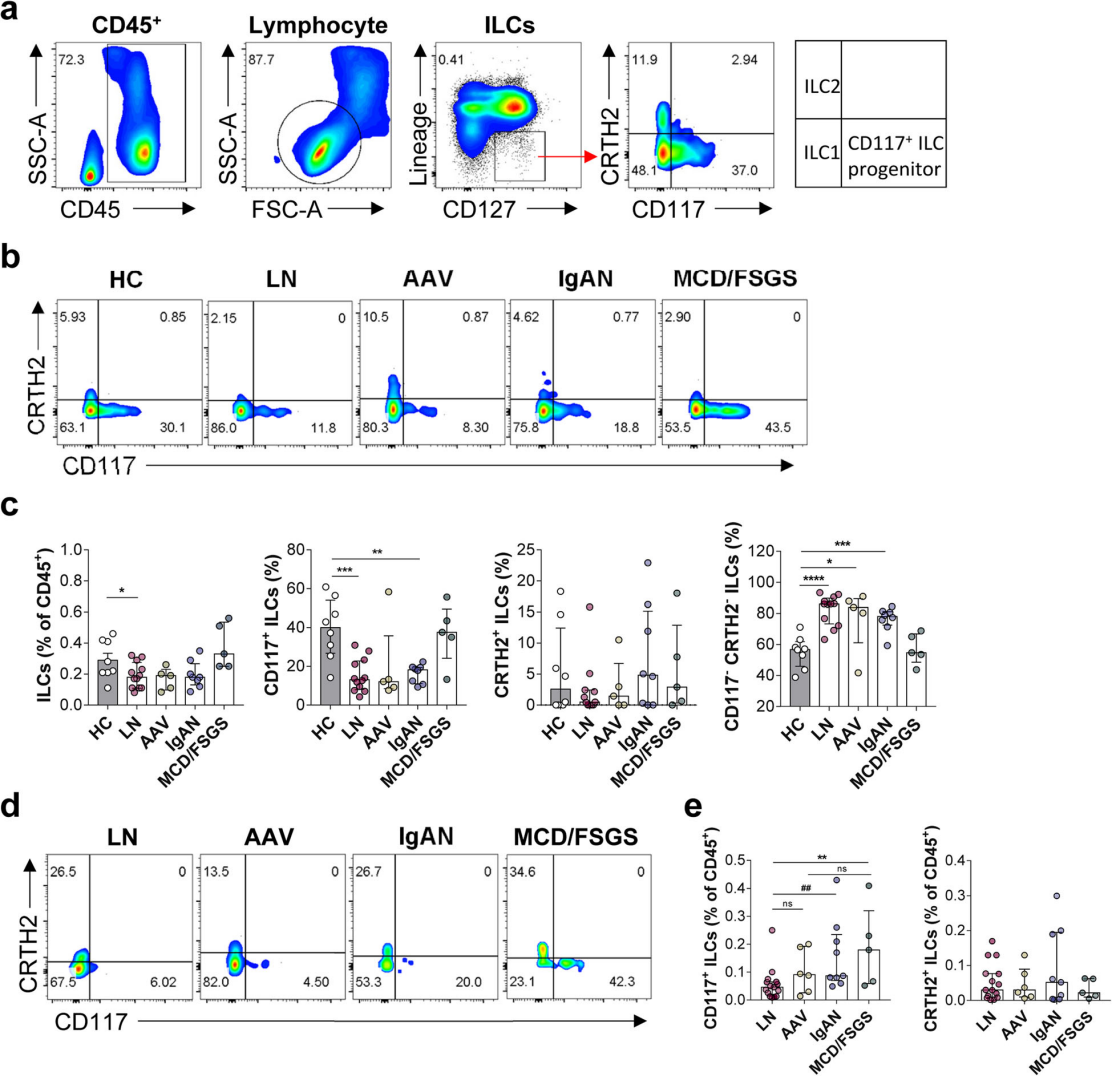
Patients with LN show reduced percentages of CD117^+^ ILCs in the circulation and in renal tissue. **a** Gating strategy for blood ILCs. **b** Representative flow cytometry plots of ILC subsets within the PBMC. **c** Percentage of total ILCs (CD45^+^ Lin^−^ CD127^+^), CD117^+^ ILCs, CRTH2^+^ ILCs, and CD117^−^/CRTH2^−^ ILCs within the PBMC from HC (*n* = 8), LN (*n* = 12), AAV (*n* = 5), IgAN (*n* = 8), and MCD/FSGS (*n* = 5). **d** Representative flow cytometry plots of ILC subsets in urine. **e** Percentage of CD117^+^ ILCs and CRTH2^+^ ILCs in urine from patients with LN (*n* = 15), AAV (*n* = 6), IgAN (*n* = 9), and MCD/FSGS (*n* = 5). *P < 0.05; **P < 0.01; ***P < 0.001 (Student’s *t* test). ^##^*P* < 0.01 (Mann-Whitney test). ILC, innate lymphoid cell; PBMC, peripheral blood mononuclear cell; HC, healthy control; LN, lupus nephritis; AAV, ANCA-associated vasculitis; IgAN, IgA nephropathy; MCD, minimal change disease; FSGS, focal segmental glomerulosclerosis

To determine the distribution of ILCs in patients with LN, we recruited 16 patients with LN, 20 with other renal diseases, and eight HC (Table 1). Blood ILCs were identified as CD45^+^Lin^−^CD127^+^ lymphocytes. These ILCs were further subdivided according to the expression of surface markers; CRTH2 for ILC2s, CD117 for ILC3s and/or progenitor of ILCs, and CRTH2^−^/CD117^−^ for ILC1s (Fig. 1a, b). The percentage of total ILCs within the CD45^+^ cell population from patients with LN was significantly lower than that in HC (*P* = 0.0481) (Fig. 1c). We noted that CD117^+^ ILCs in most patients with autoimmune kidney diseases showed reduced numbers (LN (*P* = 0.0003), AAV (*P* = 0.0877), and IgAN (*P* = 0.0014)), but not in MCD or FSGS. By contrast, the percentage of CD117^−^/CRTH2^−^ ILC1s in patients with autoimmune kidney diseases (LN, AAV, and IgAN) except MCD/FSGS was higher than that in HC.

Although we observed changes in the proportions of ILCs in the blood of patients with autoimmune kidney diseases including LN, they could not reflect local or kidney-specific immune responses completely. Therefore, we obtained urine samples from patients with LN, AAV, IgAN, and MCD/FSGS and analyzed ILCs by flow cytometry (Fig. 1d, e). ILCs were barely detectable in samples from HC because of few cell numbers in normal urine. However, the percentage of CD117^+^ ILCs in urine from LN patients was significantly lower than that in samples from patients with other renal diseases (LN vs. IgAN (*P* = 0.0034) and LN vs. MCD/FSGS (*P* = 0.0087)) (Fig. 1e). Also, we confirmed the presence of ILCs in a nephrectomized kidney by flow cytometry (Additional file 1: Figure S1) and examined CD117^+^ ILCs in renal tissue from HC and LN by immunofluorescence (IF) staining (Additional file 2: Figure S2). IF staining revealed that renal tissue from LN harbored fewer CD117^+^ ILCs than that from healthy control, although the CD3^+^ T cell count in LN was higher (Additional file 2: Figure S2d). It implied that not only circulating but also intra-renal CD117^+^ ILCs decreased in patients with LN.

### ILCs from patients with LN show reduced cytokine-producing capacity

**Fig. 2:**
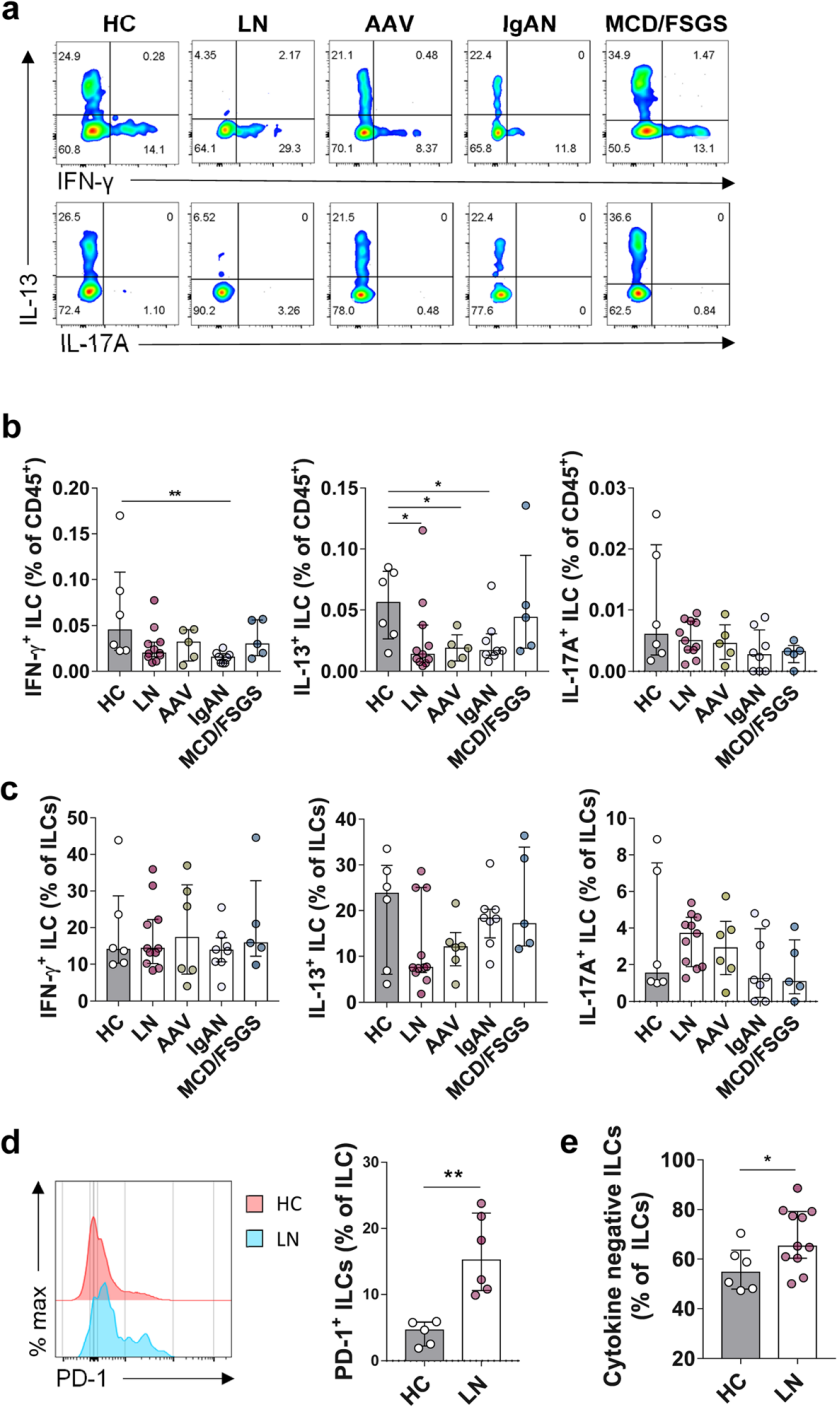
Circulating ILCs in patients with LN are dysfunctional with respect to cytokine production. **a** Representative flow cytometry plots of IFN-γ-, IL-13-, and IL-17A^+^-producing ILCs within the PBMC. **b**, **c** Percentage of IFN-γ-, IL-13-, and IL-17A^+^-producing ILCs in (**b**) CD45^+^ immune cells and (**c**) total ILCs from HC (*n* = 6), LN (*n* = 11), AAV (*n* = 5), IgAN (*n* = 8), and MCD/FSGS (*n* = 5). **d** Representative histogram plot of PD-1 expression of circulating ILCs from HC and LN, and percentage of PD-1^+^ ILCs within PBMC compared between HC (*n* = 5) and LN (*n* = 6). **e** Percentage of cytokine-negative ILCs within PBMC from HC (*n* = 6) and LN (*n* = 11). *P < 0.05; **P < 0.01 (Student’s *t* test)

To better understand the pathophysiologic function of ILCs in LN, the percentage of IFN-γ-, IL-13-, and IL-17A-producing circulating ILCs was compared with other kidney diseases (AAV, IgAN, and MCD/FSGS), and HC by flow cytometry (Fig. 2a–c). Blood ILCs from patients with LN produced less cytokines, especially IL-13, than ILCs from HC (Fig. 2b, c). The percentage of cytokine-negative ILCs, which do not produce IFN-γ, IL-13, or IL-17A, was higher in those with autoimmune kidney diseases, including LN, than in HC (Fig. 2e). ILCs from LN patients also showed higher expression of PD-1 than those from HC, suggesting that those cytokine-negative ILCs were “exhausted ILCs” (Fig. 2d). Especially, the PD-1-producing ILCs showed the characteristics of cytokine-negative ILCs (Additional file 5: Figure S5).

We noted that circulating CD117^+^ ILCs, in patients with LN (Fig. 1c), were significantly lower than that in HC; however, there was no difference in the percentage of IL-17-producing ILCs (which are conventionally regarded as IL-17A^+^ ILC3s) between HC and patients with renal disease, including LN (HC vs. LN, *P* = 0.1405). Instead, IFN-γ- and IL-13-producing ILCs (ILC1s and ILC2s, respectively) from patients with LN and other autoimmune kidney diseases were lower than in HC (Fig. 2b). Taken together, these data show that the LN microenvironment induces dysfunction of ILCs showing increased expression of PD-1.

### The percentage of CD117^+^ ILCs and cytokine-producing ILCs in LN patients with stable state is higher than in those with active state

**Fig. 3:**
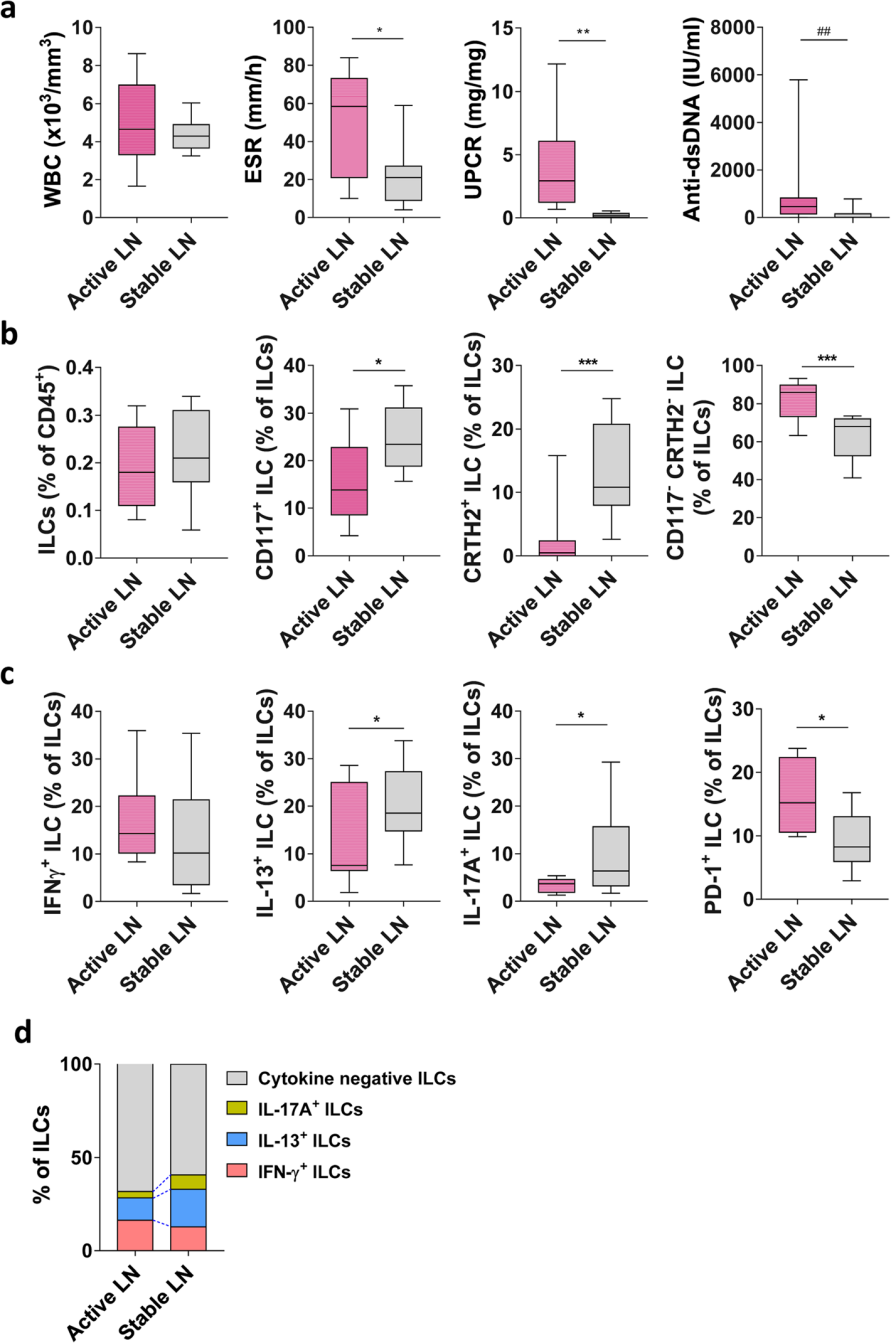
Patients with stable LN have higher numbers of circulating CD117^+^ ILCs and cytokine-producing ILCs. **a** Differences in clinical parameters of those with active and stable LN. **b** Changes in total ILCs, CD117^+^ ILCs, CRTH2^+^ ILCs, and CD117^−^/CRTH2^−^ ILCs are shown as min. to max. Box-and-whisker plots (line represents the median). **c**, **d** Changes in IFN-γ^+^, IL-13^+^, IL-17A^+^, PD-1^+^ ILCs, and cytokine-negative ILCs in PBMC from those with active LN (*n* = 12) or stable LN (*n* = 10). *P < 0.05; **P < 0.01; ***P < 0.001; ****P < 0.0001 (Student’s *t* test). ^##^*P* < 0.01 (Mann-Whitney test)

To better understand the implications of the altered percentages of CD117^+^ ILCs in those with LN, we compared the numbers of circulating ILCs in LN patients with the stable state with those in patients with the active state. We found significant differences in ESR (erythrocyte sedimentation rate), UPCR (urine protein to creatinine ratio), and anti-dsDNA levels between the two groups (Fig. 3a). Patients with stable LN had higher numbers of CD117^+^ ILCs and CRTH2^+^ ILCs than those with active LN (Fig. 3b). Consistent with this, we found that the numbers of IL-13- and IL-17A-producing ILCs were higher in those with stable LN (Fig. 3c, d). Finally, patients with stable LN had fewer exhausted ILCs, which expressed higher levels of PD-1 and failed to produce cytokines (Fig. 3c, d). These results imply that the reduced percentage of dysfunctional exhausted ILCs seen in those with active LN recovers as the disease becomes stable.

### A reduced percentage of CD117^+^ ILC progenitors and functional ILCs reflects disease activity

**Fig. 4:**
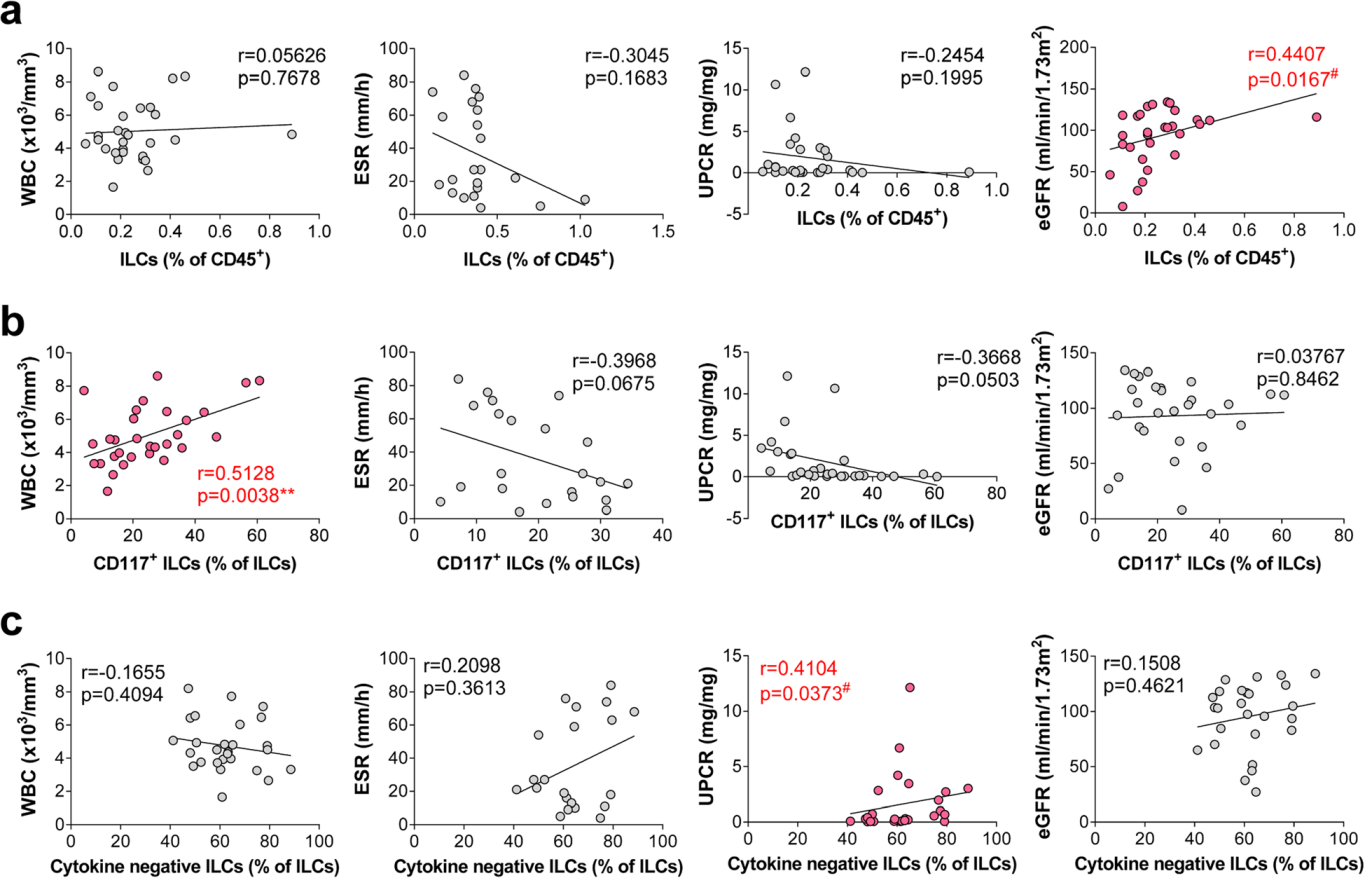
Circulating ILCs correlate with clinical parameters that reflect LN disease activity. Correlation between the percentage of (**a**) total ILCs, (**b**) CD117^+^ ILCs, and (**c**) cytokine-negative ILCs within PBMC, and clinical parameters (WBC counts, UPCR, and eGFR). **P < 0.01 (Pearson correlation). ^#^*P* < 0.01 (Spearman correlation). WBC, white blood cell; UPCR, urine protein to creatinine ratio; eGFR, estimated glomerular filtration rate

Next, we examined the correlation between the total number of ILCs (Fig. 4a), CD117^+^ ILC progenitors (Fig. 4b), cytokine-negative ILCs (Fig. 4c), and clinical parameters that reflect disease activity. Here, we found a positive correlation between CD117^+^ ILC progenitors and white blood cells (WBC; *r* = 0.5128, *P* = 0.0038), indicating a reduced number of ILCs in cases of active LN accompanying with leukopenia [23]. CD117^+^ ILCs showed a negative correlation with ESR (*r* = − 0.3968, *P* = 0.0675) and UPCR (*r* = − 0.3668, *P* = 0.0503). In addition, the percentage of cytokine-negative ILCs correlated positively with clinical indicators of kidney function (UPCR; *r* = 0.4104, *P* = 0.0373). We also observed a significant reduction in the numbers of CD117^+^ ILCs in urine as LN disease activity increased (activity index; based on glomerular and tubulointerstitial changes of LN (*r* = − 0.6368, *P* = 0.0260)) (Additional file 3: Figure S3a). Taken together, these data suggest that reduced numbers of ILCs in blood and urine could be the possible biomarker of LN severity.

### The percentage of CD117^+^ ILC progenitors in the LN microenvironment is reduced through increased differentiation

**Fig. 5:**
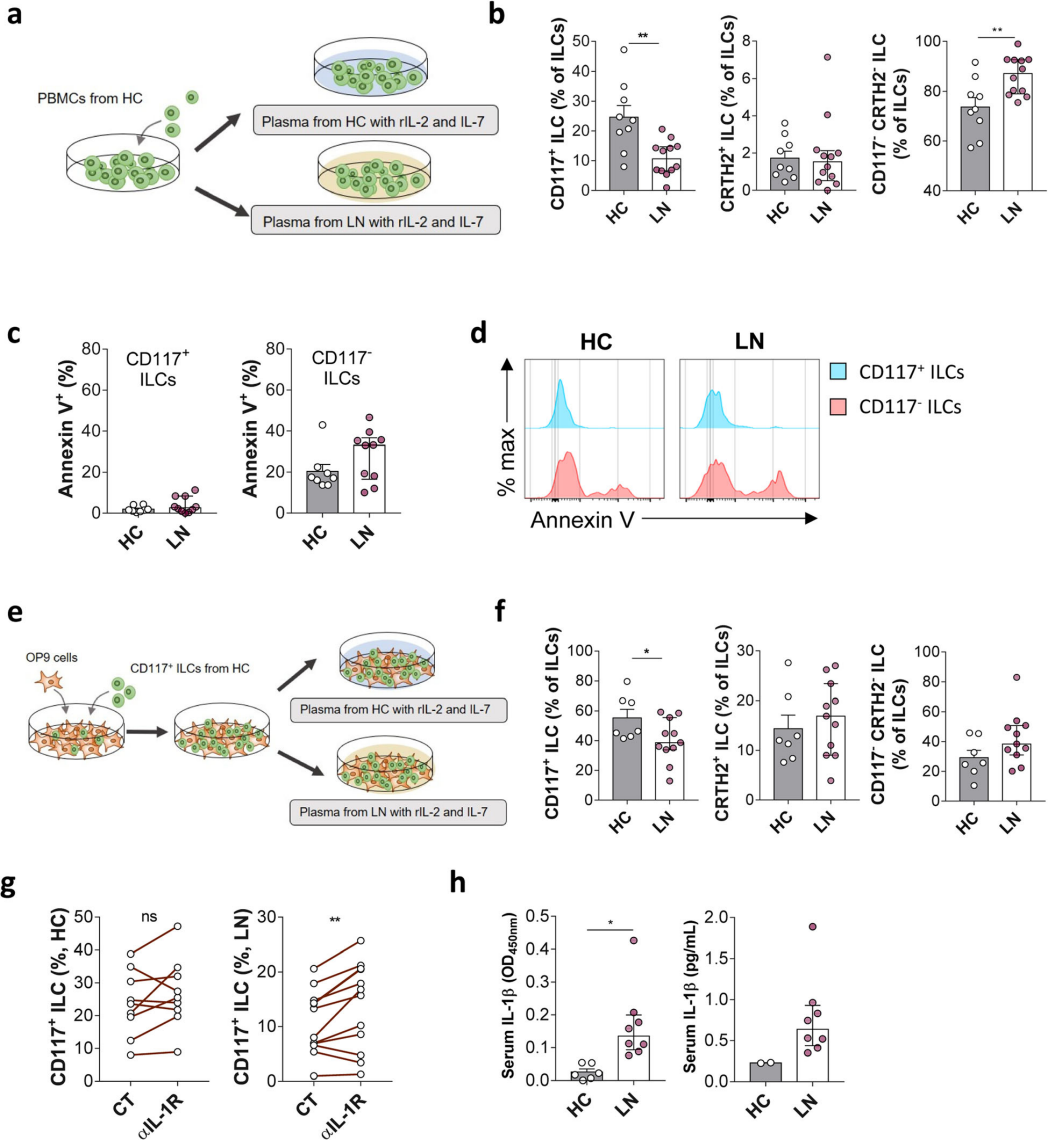
Differentiation of ILC progenitors is affected by LN microenvironment. **a** PBMCs from HC were cultured for 24 h with plasma from either HC (*n* = 9) or patients with active LN (*n* = 12). **b** Percentages of CD117^+^, CRTH2^+^, and CD117^−^ CRTH2^−^ ILCs in total ILCs were compared under both conditions. **c**, **d** Annexin V was stained to compare the apoptosis of ILCs in either conditions (*n* = 8 for HC; *n* = 10 for LN). **e** FACS-sorted CD117^+^ ILCs were cultured for 8 days in plasma from HC (*n* = 7) or patients with active LN (*n* = 11). **f** Percentages of CD117^+^, CRTH2^+^, and CD117^−^ CRTH2^−^ ILCs in CD45^+^ immune cells and total ILCs were compared between two culture systems. **g** The effect of blocking of IL-1 receptor was compared in HC (*n* = 9) and LN (*n* = 12) plasma-cultured condition as in Fig. 5a. **h** Serum IL-1β level was quantified for HC and LN, presented in optical density at 450 nm (OD_450nm_) and concentration (pg/mL). *P < 0.05 (Student’s *t* test)

Recent studies show that circulating CD117^+^ ILCs might be ILC progenitors rather than ILC3 cells [9, 24]. This, combined with observed patterns in the numbers of IL-17A^+^ ILCs and CD117^+^ ILCs in LN, led us to assume that the reduced percentage of CD117^+^ ILCs may be due to differentiation into other subsets. To verify this, we cultured PBMCs from HC (pre-cultured blood ILCs shown in Additional file 4: Figure S4) with plasma from LN patients to mimic the LN microenvironment in vitro (Fig. 5a). The percentage of CD117^+^ ILC progenitors fell even after culture with healthy plasma; however, this fall was more pronounced after culture with LN plasma (Fig. 5b). By contrast, the percentage of CD117^−^ CRTH2^−^ ILCs increased when PBMCs were cultured with plasma from patients with LN (Fig. 5b). However, there is a possibility that the decrease in CD117^+^ ILCs is due to increased cell death in the LN environment. When we stained ILCs with Annexin V, the apoptosis marker (Fig. 5c, d), cell death was not increased in CD117^+^ ILCs or CD117^−^ ILCs. Therefore, it excludes the possibilities that cell death caused the reduction of CD117^+^ ILCs, which also implied the progenitor-like characteristics of CD117^+^ ILCs. To further clarify the hypothesis that increased differentiation of CD117^+^ ILC progenitors is triggered by soluble factors in LN plasma, we isolated CD117^+^ ILC progenitors from healthy blood and cultured them with OP9 stromal cells (Fig. 5e). The percentage of CD117^+^ ILC progenitors fell, but that of other ILC subsets increased when exposed to LN plasma (Fig. 5f).

Next, we tried to figure out the soluble factor that contributed to the differentiation of ILC progenitors in LN. IL-1R1 (IL-1 receptor, type 1) exclusively expressed on human blood CD117^+^ ILCs than other blood ILC subsets [9]. The recent study of Smith et al. [25] showed that plasma IL-1β highly correlated with renal activity in SLE patients and we also found that serum IL-1β was elevated in LN compared to HC (Fig. 5h). In that sense, we hypothesized that higher IL-1β in active LN plasma would exert the differentiation of CD117^+^ ILCs through the IL-1R1 signaling pathway. To verify the hypothesis, we used IL-1R blocking antibody in the PBMC culture assay and compared the change of CD117^+^ ILCs and other ILC subsets (Fig. 5g). Blocking of IL-1 receptor did reverse the changes of CD117^+^ ILCs only in LN plasma (Fig. 5g).

Collectively, these data suggest that circulating CD117^+^ ILCs might be ILC progenitors capable of differentiating into other ILC subsets and that the reduction in the percentage of CD117^+^ ILCs in LN might be due to increased differentiation rather than cell death. Elevated IL-1β in LN might be one of the contributors to the differentiation of ILC progenitors in LN.

## Discussion

LN is the kidney manifestation of SLE and a major contributor to lupus-related mortality. The mechanism underlying disease initiation and progression is unclear; therefore, few treatments are available. Here, we characterized ILCs in patients with LN and found that they harbored fewer CD117^+^ ILCs. Although CD117^+^ ILCs are thought to be ILC3 cells [26], recent findings suggested that CD117^+^ ILCs are in fact ILC progenitors that can differentiate into any subset of ILCs [9, 27]. We confirmed that this is the case by exposing CD117^+^ ILCs to plasma from HC or LN patients. Therefore, we assume that changes in the numbers of each subset gave rise to the observed dynamics within the ILC population. The reduced percentage of ILC progenitors and functional ILCs eventually made the ILCs less functional, although the underlying mechanism requires further studies (Additional file 6: Figure S6).

Two recent studies reported changes in blood ILC populations in patients with SLE, rather than LN. With an increase in the total number of ILCs in SLE blood, Guo et al. showed that the percentage of ILC1s in peripheral blood from patients with SLE increased, but the percentage of ILC2s and ILC3s decreased [28]. Also, Kruize et al. showed that SLE patients with a high IFN signature had a higher number of ILC1s and a lower number of ILC2s than those with a low IFN signature [29]. Here, we confirmed an increase in the ILC1 (CD117^−^/CRTH2^−^ ILCs) population in those with LN; however, we did not observe increased IFN-γ production. Likewise, there were no changes in the number of ILC2s (CRTH2^+^ ILCs), although the numbers of IL-13-producing ILCs were lower in patients with LN than in HC and the numbers of ILC2s were higher in stable LN compared to active LN. Rather than examining changes in a particular ILC subset based on expression of conventional surface markers, we explored the reduction in ILC numbers in the context of changes in CD117^+^ ILC progenitors. The reduction of ILCs was also found in the kidney from lupus mouse [19].

A reduced CD117^+^ ILC population is not unique to LN; indeed, similar observations were reported for patients infected with HIV or humanized mice infected with simian immunodeficiency virus [30–33]. HIV or simian immunodeficiency virus infection is associated with reduced CD117^+^ ILC numbers in peripheral blood, tonsil, mesenteric lymph nodes, and colon [30–33]. The decrease in CD117^+^ ILCs in those infected with HIV might be due to an overall increase in cell death [31–33]; however, the evidence of cell death was not clear [30]. Kloverpris et al. showed that the number of all ILCs including CD117^+^ ILCs were decreased in HIV infection [30]. If the reduction in ILC numbers in those with HIV infection is not due to an increase in apoptosis, it is plausible that the reduced number of CD117^+^ ILC progenitors may result in the reduction in total ILC numbers, as shown in our study. From this perspective, Lim et al. transferred human CD117^+^ ILCs into BALB/c *Rag2*^*−/−*^*Il2rg*^*−/−*^*Sirpa*^*NOD*^ (BRGS) mice that are permissive for robust multi-lineage human hematopoietic stem cell engraftment; they found that CD117^+^ ILCs successfully reconstituted ILCs in various organs (lung, gut, liver, and spleen) [9]. A recent study by Nagasawa et al. succeeded in verifying the presence of CD117^+^ ILC progenitors in peripheral organs (nasal polyps) which correspond to the circulating progenitors, but with limited potential for multi-lineage differentiation [34]. These results imply that circulating CD117^+^ multi-potent ILC progenitors can differentiate into tissue-resident ILCs to maintain tissue homeostasis. In this study, the blood CD117^+^ ILCs were examined in vitro whether they could differentiate into the other subsets of ILCs in LN plasma compared to HC plasma. The differentiation of CD117^+^ ILCs was observed when they were co-cultured with LN plasma.

We also assessed a few other possibilities of reduced CD117^+^ ILCs in LN, other than the differentiation of CD117^+^ ILCs. First, there is a possibility that the decrease in CD117^+^ ILCs is due to increased cell death in the LN environment. The idea is similar to the previous study that IL-17A-producing CD117^+^ ILC3s were reduced in HIV-infected patients [31]. Zhang et al. showed that increased type 1 interferon induced apoptosis of ILC3s. Considering the level of type 1 interferon increased in SLE [35], a decrease of CD117^+^ ILCs may be due to increased apoptosis. To test this, we analyzed the apoptosis of ILCs and found that CD117^+^ ILCs were not stained with Annexin V. Therefore, it is hard to say the decrease of CD117^+^ ILCs is due to the increased cell death. Second, the proliferation capacity of CD117^+^ ILCs may be lower than that of other subsets of ILCs. When we compared the proliferative activity of CD117^+^ ILCs and CD117^−^ ILCs from HC or LN plasma-cultured condition, there was no significant difference between HC and LN (data not shown). There is, however, a limitation that short-term culture condition might be unsuitable for detecting proliferation. The last hypothesis is the trans-differentiation of CD117^+^ ILCs in response to the local environment from LN. It has been reported that not only murine ILCs but also human blood and nasal polyp derived ILC2s could trans-differentiate by stimulation of a set of cytokines (IL-1β, IL-13, and TGF-β) [36]. However, the majority of CD117^+^ ILCs shown in our data were not fully functional, cytokine-producing ILC3s (Figs. 1c and 2c). Since the trans-differentiation means the functional or phenotypical change of a differentiated ILC into another subset [37], it would not be appropriate indicating the observed phenomenon of reduced CD117^+^ ILCs in LN as the result of trans-differentiation or plasticity. Overall, it would be appropriate to explain that the reduction of CD117^+^ ILCs either in vivo or in vitro is a result of increased differentiation of the ILC progenitors in LN environment.

Given the fact that the IL-1R1 is expressed on CD117^+^ ILCs, IL-1β, which is elevated in LN plasma, was the first candidate factor to induce the differentiation of ILCs. Blocking of IL-1 receptor reversed the phenomenon of reducing percentage of CD117^+^ ILCs in LN. Considering their marginal effects on the ILC progenitors, however, further exploratory study is still needed to understand the exact mechanism of ILC biology in patients with LN.

To examine the dynamics of ILCs in inflamed LN kidney tissue in real-time, we investigated ILCs in urine. Although the number of ILCs in urine was pretty small, we found that patients with LN had lower numbers of urine CD117^+^ ILCs than those with other kidney diseases, including AAV and IgAN which also showed reduction of those cells in blood as in LN. The finding that only LN ILCs showed a significant correlation with various markers of nephritis might be due to marked changes in the cell numbers in the target organ. The use of urine immune cells as a surrogate for those in renal tissue was thoroughly verified by the recent study by Arazi et al [38]. Immunofluorescence staining of intra-renal CD117^+^ ILCs confirmed their presence in a healthy kidney; however, they were barely detected in LN. It is still difficult to verify whether the reduced number of CD117^+^ ILCs in LN urine is due to a reduction in the number of ILC progenitors.

Finally, we compared the subset distribution and function of ILCs from patients with active and stable LN. We noted that the reduction in ILC numbers in those with active LN recovered when the disease stabilized. Furthermore, we assessed the correlation between ILCs and various clinical parameters. Changes of eGFR, WBC count [23], and proteinuria amount, all of which reflect LN activity, correlated significantly with a reduction in the total number of ILCs and in the number of CD117^+^ ILCs. In addition, we found that as the number of exhausted ILCs increased, disease status worsens. Reduced cytokine production by ILCs was not limited to a particular subset. This phenomenon could be explained in several ways. First, the reduction in cytokine-producing ILCs could be linked directly to increased differentiation of CD117^+^ ILC progenitors. Decreasing numbers of ILC progenitors could affect the differentiation machinery and contribute to increased ILC dysfunction. Second, the phenomenon we found might be due to exhaustion of ILCs. Compared with T cells, few studies have reported exhaustion of ILCs [39, 40]. All of these studies were based on murine models, but all linked dysfunction of ILCs to expression of PD-1, a well-known immune inhibitory molecule [41, 42]. Indeed, circulating CD4 T cells from patients with rheumatoid arthritis show higher expression of PD-1 than those from HC [43]. We also found that expression of PD-1 by ILCs from patients with active LN was higher than that by ILCs from those with stable LN. ILCs from those with active LN produce less cytokine (especially, IL-13 and IL-17A) than those from patients with stable LN. Third, it is possible that other cytokines (not examined here) might be involved in the pathogenesis of LN. In addition to common cytokines such as IFN-γ, IL-13, and IL-17A, ILCs could secrete amphiregulin [44], GM-CSF [45], and IL-9 [46], all of which have pathogenic or protective effects under various disease conditions.

## Conclusions

In summary, we show here that LN patients harbor reduced numbers of total ILCs and CD117^+^ ILCs, and that ILCs show impaired cytokine production. The take-home points are as follows: (1) this is the first study of human ILCs to compare LN with other renal diseases, (2) urine ILCs were used as a surrogate marker for ILCs in kidney tissues, and (3) the dynamics of ILCs in those with active and stable LN was supported by in vitro experiments with ILC progenitors. A limitation of this study is that there was restricted opportunity to look into the role of ILCs in other autoimmune kidney diseases except SLE, although they also showed similar trends in circulating ILCs. This is, however, the first step to understand the role of ILCs in LN and other autoimmune kidney diseases. Considering their protective role in murine kidney, follow-up studies focusing on the role of ILCs in the pre-clinical status of these diseases when ILCs are abundant and fully functional will increase our understanding of immune reactions in LN.

## Supplementary information

**Additional file 1: Figure S1.** Phenotype and functional aspects of kidney ILCs from pathologically normal renal tissue obtained from a patient with renal cell carcinoma.

**Additional file 2: Figure S2.** Immunofluorescence staining of CD117^+^ ILCs (c, d; found as CD3^-^ CD117^+^) in renal tissue showed that they were decreased in renal tissue of (b) LN, compared to that of (a) HC. (c) DAPI (blue), CD3 (green), and CD117 (red), and merged image. (d) The number of CD3^-^CD117^+^ cells within the same area in renal tissue of HC and LN. Pink arrow: CD3^+^ T cells, white arrow: CD117^+^ ILCs, orange arrow: CD3^+^ CD117^+^ T cells. *P<0.05 (Student’s t test).

**Additional file 3: Figure S3.** Correlation between the percentage of urine ILCs ((a) CD117^+^ ILCs and (b) CRTH2^+^ ILCs) and clinical parameters including serum BUN and creatinine, activity and chronicity index. *P<0.05 (Pearson correlation).

**Additional file 4: Figure S4.** Blood ILCs from a healthy control before in vitro culture, related to Fig. 5a, b.

**Additional file 5: Figure S5.** PD-1 expressing ILCs within PBMCS did not express IFN-γ, IL-13, or IL-17A. ILCs in both (a) HC and (b) LN.

**Additional file 6: Figure S6.** Working model. In healthy kidney, cytokine producing ILCs maintain tissue homeostasis. In patients with LN, reduced numbers of ILC progenitor cells result in exhaustion of the ILCs with increased expression of PD-1. A reduction of functional ILCs might associate with increased severity of LN. Figure contains some images adapted from SMART (Servier Medical Art; http://smart.servier.com/), licensed under a Creative Common Attribution 3.0 Generic License.

## Data Availability

All data generated or analyzed during this study are included in this published article.

## Abbreviations

AAV: ANCA-associated vasculitis
BUN: Blood urea nitrogen
C3: Complement component 3
C4: Complement component 4
CRP: C-reactive protein
dsDNA: Double-stranded DNA
FSGS: Focal segmental glomerulosclerosis
HC: Healthy control
IgAN: IgA nephropathy
ILC: Innate lymphoid cell
LN: Lupus nephritis
MCD: Minimal change disease
PBMC: Peripheral blood mononuclear cell
UPCR: Urine protein to creatinine ratio
WBC: White blood cell
