# Supplementary figures and images for: Reduction of circulating innate lymphoid cell progenitors results in impaired cytokine production by innate lymphoid cells in patients with lupus nephritis
Source: Arthritis Res Ther. 2020 Mar 29;22:63. doi: 10.1186/s13075-020-2114-5 (PMC7104540; doi:10.1186/s13075-020-2114-5)

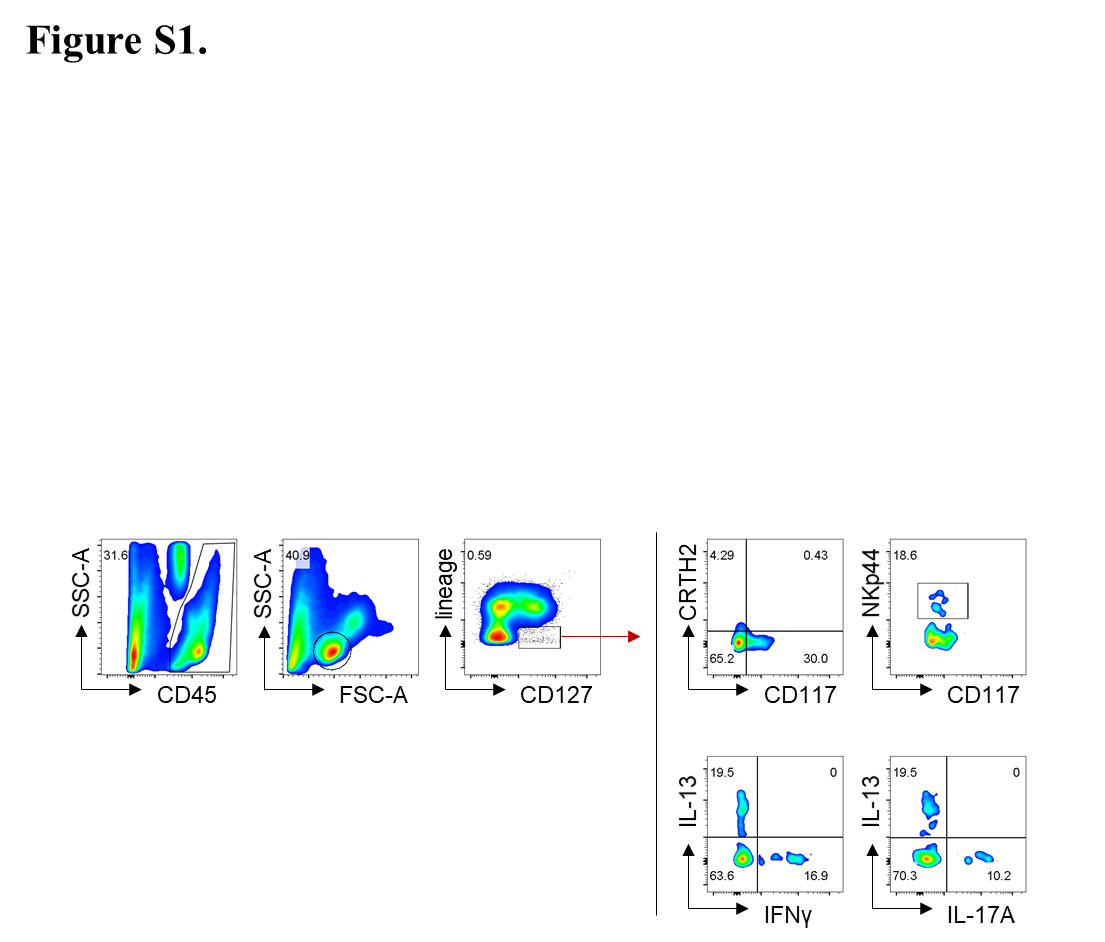

Supplement: Supplementary file 1 — Additional file 1: Figure S1. Phenotype and functional aspects of kidney ILCs from pathologically normal renal tissue obtained from a patient with renal cell carcinoma. [file 13075_2020_2114_MOESM1_ESM.docx]

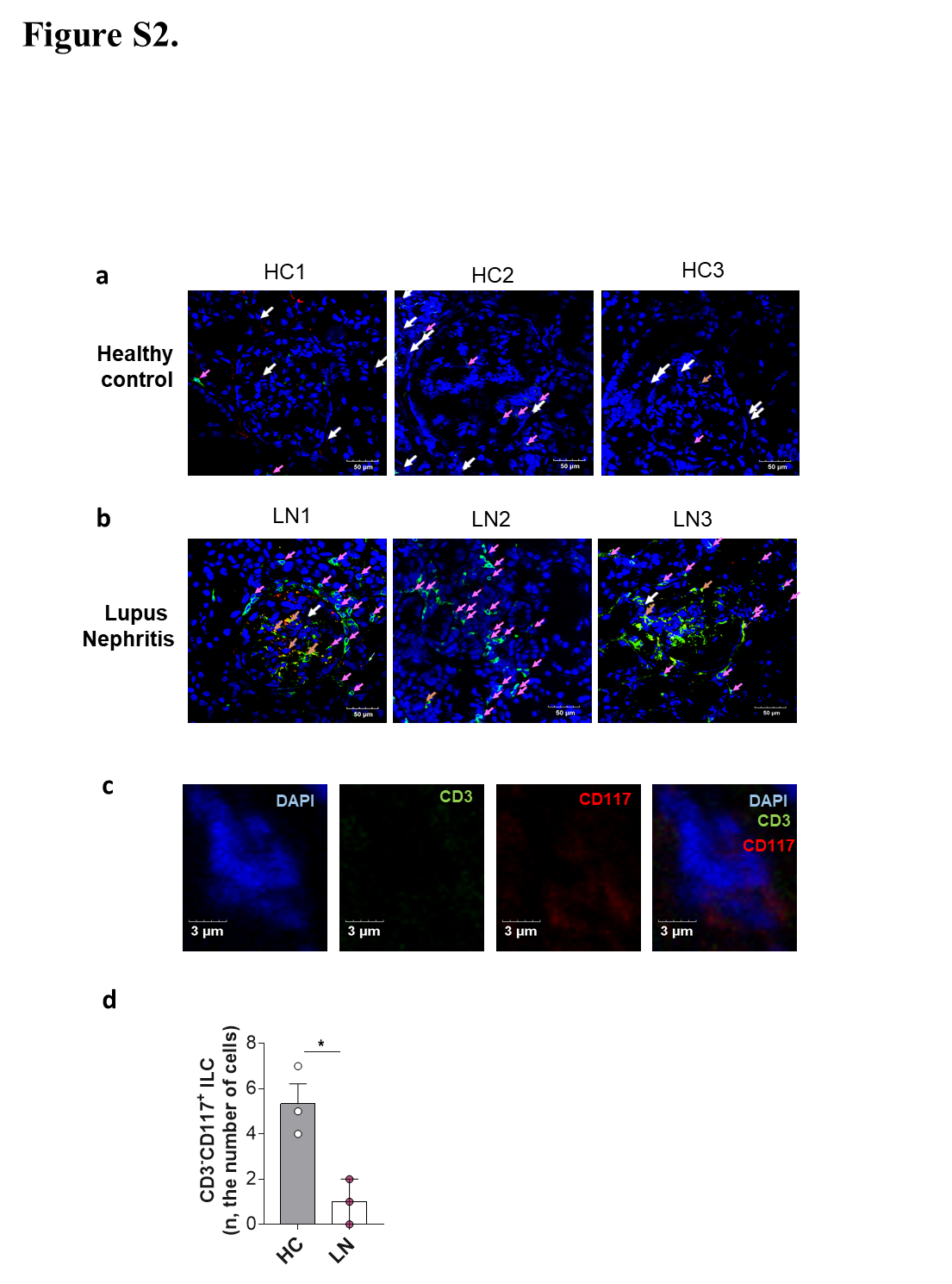

Supplement: Supplementary file 2 — Additional file 2: Figure S2. Immunofluorescence staining of CD117+ ILCs (c, d; found as CD3- CD117+) in renal tissue showed that they were decreased in renal tissue of (b) LN, compared to that of (a) HC. (c) DAPI (blue), CD3 (green), and CD117 (red), and merged image. (d) The number of CD3-CD117+ cells within the same area in renal tissue of HC and LN. Pink arrow: CD3+ T cells, white arrow: CD117+ ILCs, orange arrow: CD3+ CD117+ T cells. *P<0.05 (Student’s t test). [file 13075_2020_2114_MOESM2_ESM.docx]

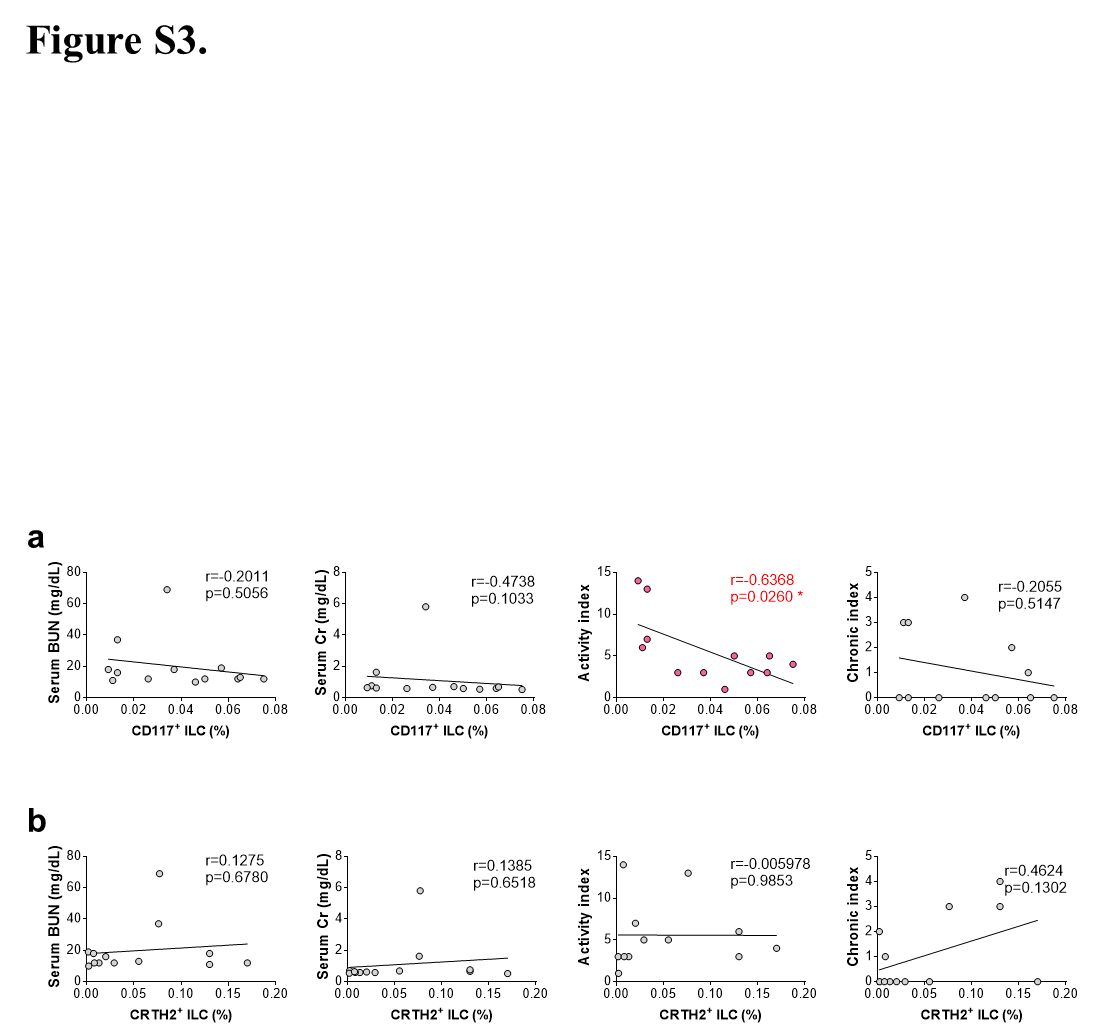

Supplement: Supplementary file 3 — Additional file 3: Figure S3. Correlation between the percentage of urine ILCs ((a) CD117+ ILCs and (b) CRTH2+ ILCs) and clinical parameters including serum BUN and creatinine, activity and chronicity index. *P<0.05 (Pearson correlation). [file 13075_2020_2114_MOESM3_ESM.docx]

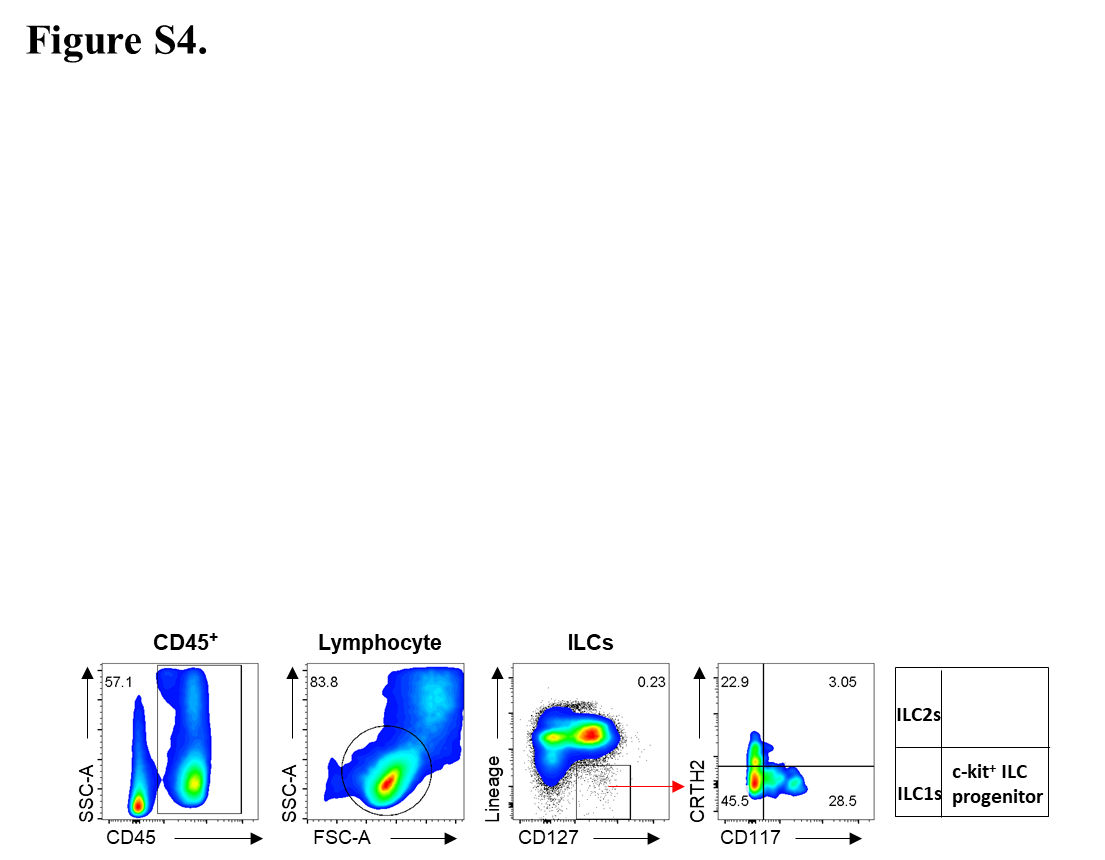

Supplement: Supplementary file 4 — Additional file 4: Figure S4. Blood ILCs from a healthy control before in vitro culture, related to Fig. 5a, b. [file 13075_2020_2114_MOESM4_ESM.docx]

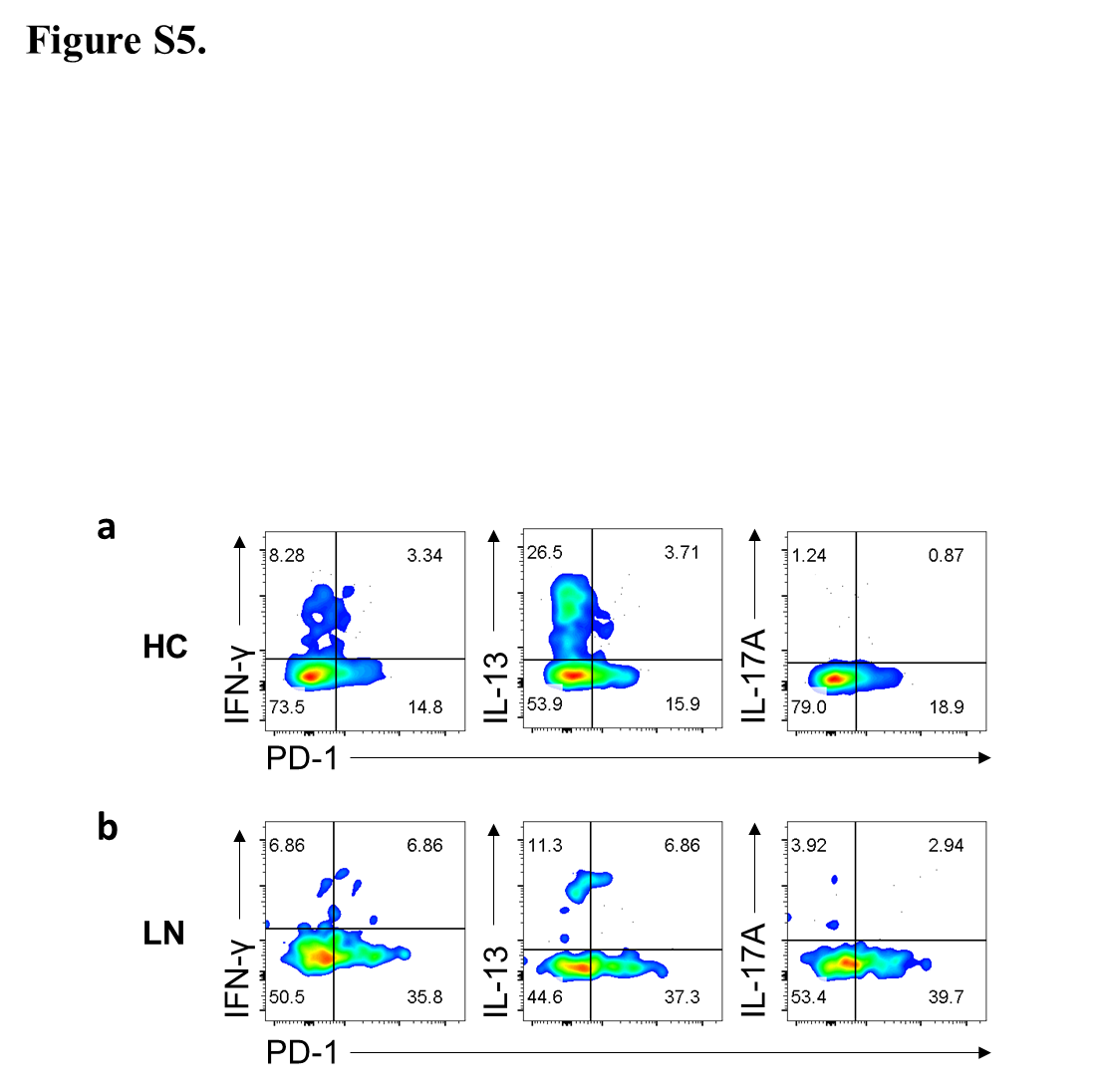

Supplement: Supplementary file 5 — Additional file 5: Figure S5. PD-1 expressing ILCs within PBMCS did not express IFN-γ, IL-13, or IL-17A. ILCs in both (a) HC and (b) LN. [file 13075_2020_2114_MOESM5_ESM.docx]

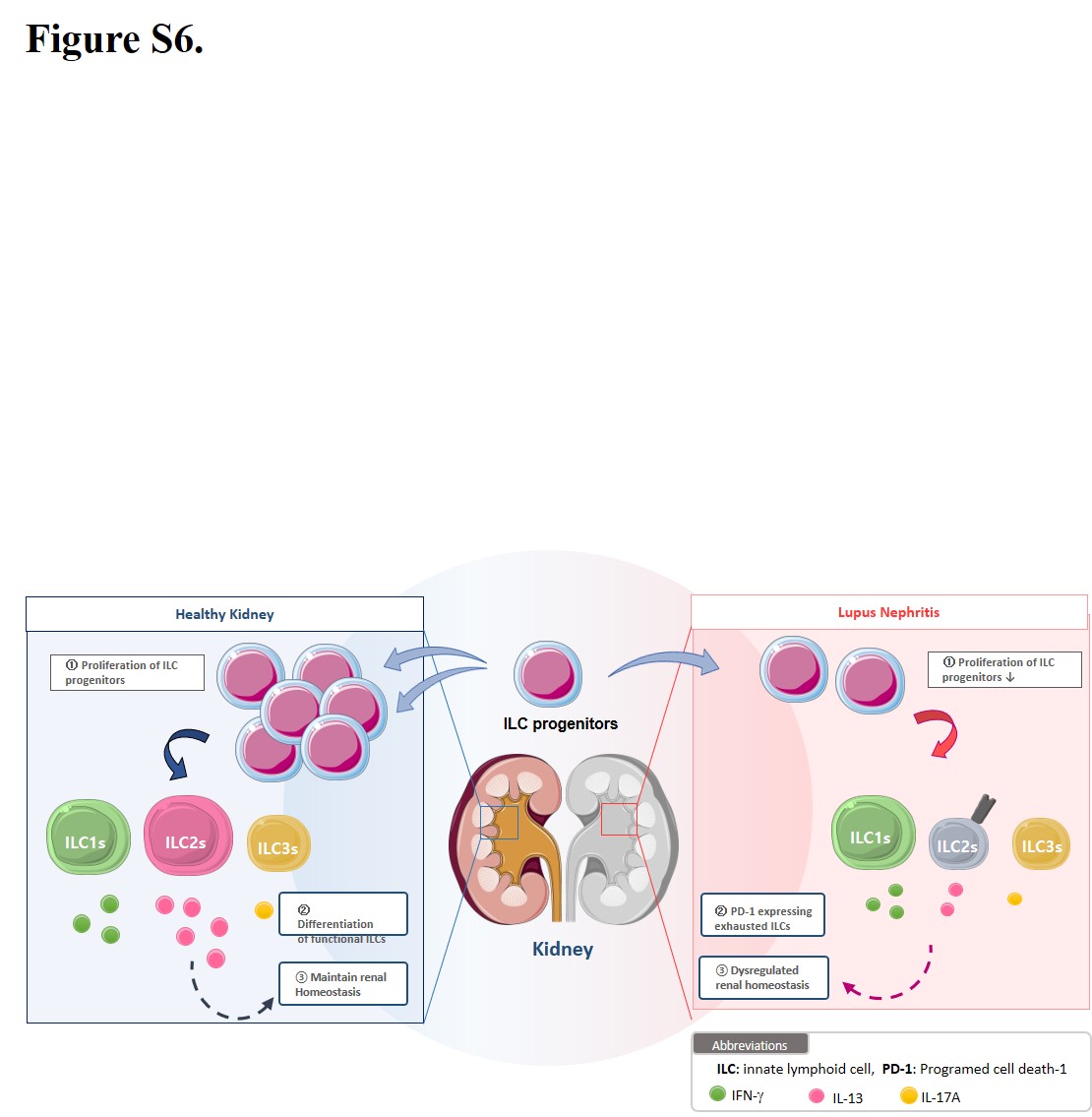

Supplement: Supplementary file 6 — Additional file 6: Figure S6. Working model. In healthy kidney, cytokine producing ILCs maintain tissue homeostasis. In patients with LN, reduced numbers of ILC progenitor cells result in exhaustion of the ILCs with increased expression of PD-1. A reduction of functional ILCs might associate with increased severity of LN. Figure contains some images adapted from SMART (Servier Medical Art; http://smart.servier.com/), licensed under a Creative Common Attribution 3.0 Generic License. [file 13075_2020_2114_MOESM6_ESM.docx]
